# Multicountry Spread of Influenza A(H1N1)pdm09 Viruses with Reduced Oseltamivir Inhibition, May 2023–February 2024

**DOI:** 10.3201/eid3007.240480

**Published:** 2024-07

**Authors:** Mira C. Patel, Ha T. Nguyen, Philippe Noriel Q. Pascua, Rongyuan Gao, John Steel, Rebecca J. Kondor, Larisa V. Gubareva

**Affiliations:** Centers for Disease Control and Prevention, Atlanta, Georgia, USA

**Keywords:** Influenza, antiviral, neuraminidase inhibitors, oseltamivir, reduced inhibition, substitution, baloxavir, phenotypic testing, viruses, influenza A(H1N1)pdm09, H1N1, pH1N1, antimicrobial resistance, United States

## Abstract

Since May 2023, a novel combination of neuraminidase mutations, I223V + S247N, has been detected in influenza A(H1N1)pdm09 viruses collected in countries spanning 5 continents, mostly in Europe (67/101). The viruses belong to 2 phylogenetically distinct groups and display ≈13-fold reduced inhibition by oseltamivir while retaining normal susceptibility to other antiviral drugs.

Three classes of direct-acting antivirals targeting the influenza virus matrix protein 2 (M2) ion channel, neuraminidase (NA), or polymerase cap-dependent endonuclease (CEN) are approved to treat influenza in many countries (*1*). Although most seasonal influenza viruses are susceptible to NA and CEN inhibitors, emergence of antiviral-resistant variants is a public health concern because of widespread resistance to M2 inhibitors and possibilities of similar resistance developing for other antiviral drugs (*2*). Oseltamivir, an NA inhibitor, is the drug most prescribed for influenza (*2*). The NA amino acid substitution H275Y, acquired spontaneously or after drug exposure, confers resistance to oseltamivir. Oseltamivir-resistant influenza A(H1N1) viruses with H275Y emerged first in Europe during 2007–2008 and rapidly spread worldwide (*3*). However, they were displaced by influenza A(H1N1)pdm09 (pH1N1), the swine-origin virus that caused the 2009 pandemic (*4*).

Monitoring oseltamivir susceptibility is a priority for the World Health Organization Global Influenza Surveillance and Response System (WHO-GISRS). In addition to H275Y, many NA substitutions in N1 subtype viruses are suspected of reducing oseltamivir susceptibility (*5*). Although there are no established criteria for determining clinically relevant oseltamivir resistance based on phenotypic testing, for surveillance purposes, influenza A viruses tested in NA inhibition assays are classified as displaying reduced inhibition if they have a 50% inhibitory concentration (IC_50_) 10-100–fold higher or as highly reduced inhibition if IC_50_ >100-fold higher than that of a reference (*6*).

## The Study

The Centers for Disease Control and Prevention (CDC) monitors antiviral susceptibility of viruses submitted to the national surveillance system and those collected in other countries. Nearly all influenza-positive samples undergo next-generation sequencing. We analyzed NA sequences of submitted viruses for substitutions previously associated with reduced susceptibility (*5*), tested the viruses in an NA inhibition assay, and compared IC_50_s with a reference IC_50_ to determine inhibition levels (*7*).

During May 2023–February 2024, we analyzed 2,039 pH1N1 viruses from the United States (n = 1,274) and 38 other countries (n = 765). Four had the H275Y substitution, indicating low frequency of oseltamivir resistance. Analysis revealed NA substitution I223V in 18 and S247N in 15 viruses; those substitutions confer mildly elevated oseltamivir IC_50_ (<10-fold). We also detected 17 viruses carrying both substitutions, I223V + S247N (Table 1; Appendix Table 1). As expected, single mutants exhibited normal inhibition by oseltamivir and other NA inhibitors in NA inhibition assay (Table 2). The 6 viruses with I223V + S247N displayed 13- to 16-fold reduced inhibition for oseltamivir and normal inhibition (<4-fold) for other NA inhibitors (Table 2; Appendix Table 2). Both single and dual mutants remained susceptible to the CEN inhibitor baloxavir (Table 2).

**Table 1:** Influenza A(H1N1)pdm09 viruses with amino acid substitutions in neuraminidase that may affect inhibition by oseltamivir*

NA substitution†	No. viruses with NA substitution	Viruses collected from countries (no. in each)
H275Y	4	Argentina (1),‡ Panama (1), USA (2)
I223V	18	Abu Dhabi (5), Bangladesh (5), Bahrain (2), Canada (1), Costa Rica (1), USA (4)
S247N	15	Abu Dhabi (2), Bhutan (3), Brazil (1), Canada (1), Hong Kong (1), Oman (1), USA (6)
I223V + S247N§	17	Bangladesh (11), Hong Kong (1), Maldives (1), Niger (3), USA (1)

*Next-generation sequencing–based virologic surveillance conducted at the Centers for Disease Control and Prevention, May 2023–February 2024. Summary of NA amino acid substitutions assessed for their effects on inhibition by NA inhibitors prepared by members of the World Health Organization Antiviral Working Group (*5*) was used as a reference for NA sequence analysis. A total of 2,039 NA sequences of influenza A(H1N1)pdm09 viruses (duplicate sequences excluded; n = 1,274 viruses collected from the United States and n = 765 from 38 other countries) were analyzed to screen for previously listed or suspected NA amino acid substitutions. Detailed information about those viruses is provided in Appendix Table 1). NA, neuraminidase. †According to a subtype-specific NA amino acid numbering system. ‡This virus also contains NA-S247N. Virus was not recovered in cell culture. §Combination of I223V + S247N was not reported previously.

**Table 2:** Antiviral susceptibility of available influenza A(H1N1)pdm09 virus isolates with NA-I223V or NA-S247N or NA-I223V + S247N substitutions*

NA substitution†	No. virus isolates tested	NA inhibitors IC_50_, nM, average ± SD (fold)‡				CEN inhibitor baloxavir EC_50_, nM, average ± SD (fold)§
		Oseltamivir	Zanamivir	Peramivir	Laninamivir	
Test viruses						
I223V	9	0.71 ± 0.17 (3)	0.23 ± 0.03 (1)	0.08 ± 0.01 (1)	0.25 ± 0.01 (1)	1.15 ± 0.15 (2)
S247N	6	0.82 ± 0.16 (4)	0.34 ± 0.05 (2)	0.19 ± 0.01 (3)	0.55 ± 0.03 (3)	1.46 ± 0.62 (2)
I223V + S247N	6	2.71 ± 0.20 (13)	0.49 ±0.02 (3)	0.29 ± 0.01 (4)	0.64 ± 0.02 (3)	0.67 ± 0.14 (1)
Reference						
Median IC_50_/EC_50_ 2022–2023	253	0.21	0.19	0.07	0.21	0.70

*Next-generation sequencing–flagged influenza A(H1N1)pdm09 viruses were propagated in MDCK-SIAT1 cells, followed by sequence confirmation of virus isolates. Susceptibility of available virus isolates with indicated single or dual NA substitution(s) were tested for NA inhibitors (oseltamivir, zanamivir, peramivir, and laninamivir) by using Centers for Disease Control and Prevention–standardized fluorescence-based NA inhibition assay and baloxavir by using a cell-based Influenza replication inhibition neuraminidase-based assay. Virus isolates were tested against each antiviral in at least 2–3 independent tests, and average ± SD of IC_50_s/EC_50_s is shown. Fold changes in IC_50_s/EC_50_s of test viruses were calculated compared with median IC_50_s/EC_50_s of influenza A(H1N1)pdm09 viruses tested during the 2022–23 influenza season (October 1, 2022–September 30, 2023). CEN, cap-dependent endonuclease; EC_50_, 50% effective concentration; IC_50_, 50% inhibitory concentration; NA, neuraminidase. †According to a subtype-specific NA amino acid numbering system. ‡Fold changes were interpreted according to World Health Organization classification criteria for type A: <10-fold, normal inhibition; 10–100-fold, reduced inhibition; >100-fold, highly reduced inhibition. §Fold changes were interpreted according to arbitrary criteria, according to which >3-fold increase in EC_50_ of test virus compared with reference EC_50_ is defined as reduced susceptibility to baloxavir.

**Figure 1:**
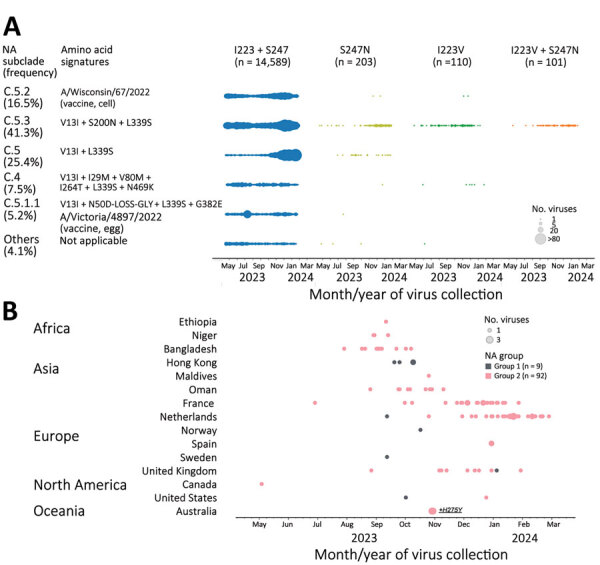
Detection of influenza A(H1N1)pdm09 viruses with dual NA-I223V + S247N substitutions through NA inhibitors susceptibility surveillance conducted by the Centers for Disease Control and Prevention and analysis of available sequences (GISAID EpiFlu, https://www.gisaid.org, accessed March 11, 2024), May 2023–February 2024. A total of 15,003 NA sequences of pH1N1 viruses (duplicate sequences excluded: 2,039 from Centers for Disease Control and Prevention and the remaining 12,964 from GISAID EpiFlu) were analyzed to screen for amino acid substitutions at residues 223 and 247. A) Introduction of single substitution (I223V or S247N) or dual substitutions (I223V + S247N) across NA subclades of pH1N1 viruses circulating during May 2023–February 2024. Amino acid signatures of NA subclades are shown in comparison to A/Wisconsin/67/2022, the Northern Hemisphere 2023–2024 vaccine cell prototype virus for the pH1N1 component. Vaccine viruses, A/Wisconsin/67/2022 and A/Victoria/4897/2022 (Northern Hemisphere 2023–2024 vaccine egg prototype virus), represented NA subclades C.5.2 and C.5.1.1, respectively. C.5.3 was the subclade most abundantly sequenced (41.3% frequency), followed by other minor subclades: C.5 (25.4%), C.5.2 (16.5%), C.4 (7.5%), C.5.1.1 (5.2%), and others (4.1%). Most viruses with single S247N substitution belonged to dominant NA subclade C.5.3 and minor subclade C.5. Conversely, most viruses with single I223V substitution and all viruses with dual I223V + S247N substitutions belonged to NA subclade C.5.3. B) Spatiotemporal distribution of dual mutant viruses. Dual mutants were divided into 2 groups based on their NA sequence difference. Group 1 shared additional substitution R257K not found in group 2. The small group 1 had 9 dual mutants, and the large group 2 had 92 dual mutants. The first dual mutant belonging to group 2 was collected in Canada at the end of May 2023, and most dual mutants were collected between September 2023 and February 2024. Two dual mutant viruses collected in Australia also contained NA-H275Y. NA, neuraminidase.

The dual mutants were collected in the United States and 4 other countries during August–November 2023 (Table 1; Appendix Table 1), which prompted us to explore when dual mutants emerged and how broadly single or dual mutants were circulating worldwide. Analysis of available NA sequences from the GISAID EpiFlu database (https://www.gisaid.org) revealed that pH1N1 viruses with single mutation I223V (n = 110) or S247N (n = 203) were found in many countries (Figure 1, panel A). In addition to the 17 viruses we sequenced, 84 additional dual mutants were identified (total n = 101). Together, they were collected in 15 countries spanning 5 continents (Africa, Asia, Europe, North America, and Oceania) (Figure 1, panel B). The first dual mutant was collected from Canada in May 2023, and the latest were collected from 4 countries (France, the Netherlands, Spain, and the United Kingdom) during January–February 2024. Most dual mutants were detected in the Netherlands (n = 30), France (n = 24), Bangladesh (n = 11), Oman (n = 9), and the United Kingdom (n = 9); fewer were found in Hong Kong (n = 4), Niger (n = 3), Australia (n = 2), Spain (n = 2), and the United States (n = 2). One dual mutant each was detected in Canada, Ethiopia, Maldives, Norway, and Sweden (Figure 1, panel B; Appendix Tables 1, 3).

**Figure 2:**
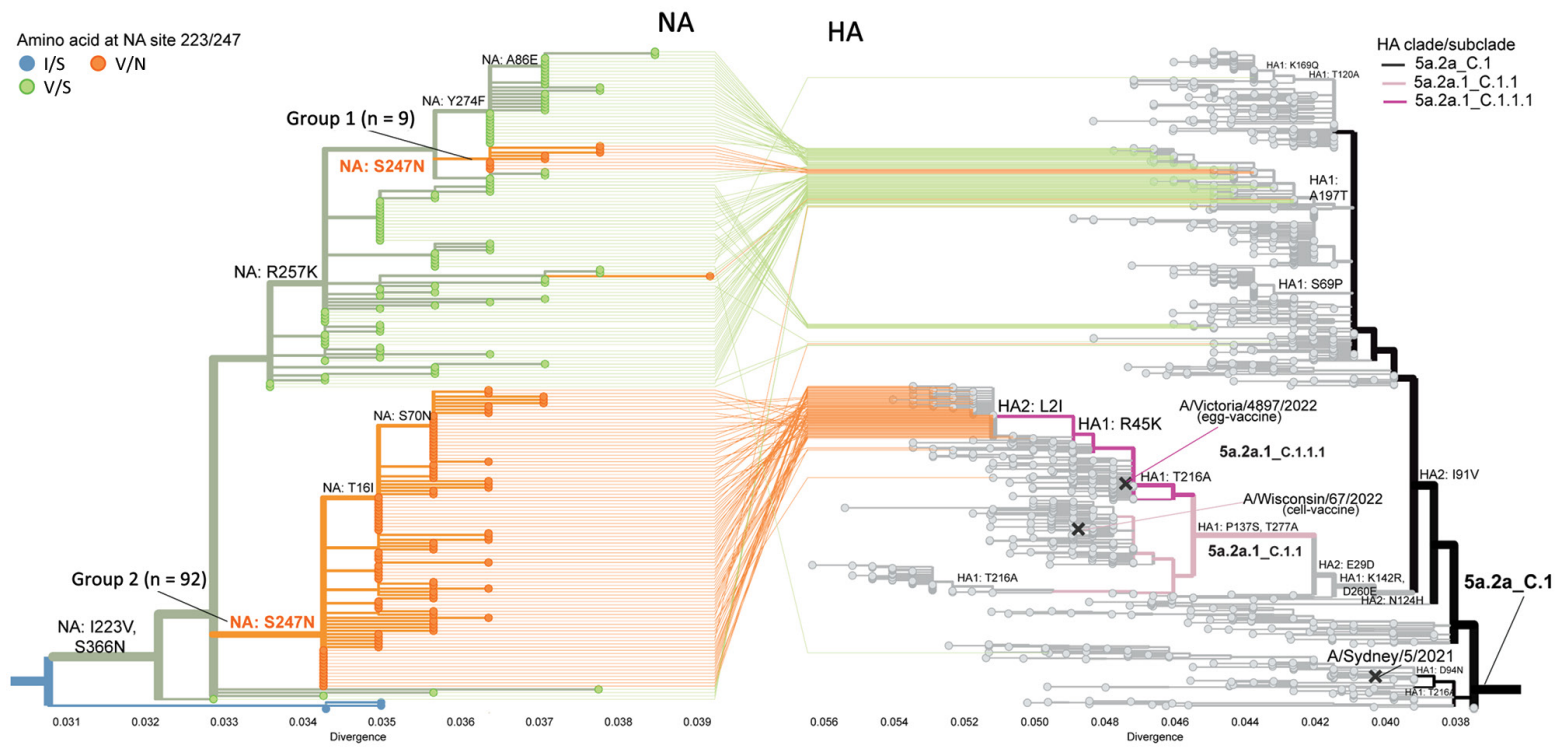
Tanglegram showing influenza A(H1N1)pdm09 phylogenies for NA gene (left) and HA gene (right) from susceptibility surveillance conducted by the Centers for Disease Control and Prevention and analysis of available sequences (GISAID EpiFlu, https://www.gisaid.org, accessed March 11, 2024), May 2023–February 2024. The NA-HA tangle tree was constructed by using Nextclade (*8*) and visualized by Auspice (https://auspice.us). NA phylogenetic tree is zoomed to show only subclade C.5.3 with 2 groups of dual I223V + S247N mutants. All dual mutant viruses shared >6 NA amino acid substitutions (V13I, S200N, I223V, S247N, L339S, S366N) compared with vaccine prototype virus A/Wisconsin/67/2022. Group 1 shared an additional substitution R257K not observed in group 2. Tree tips colored in blue indicate viruses with wild type amino acids at residues 223 and 247 (i.e., I223 and S247); green shows single I223V mutants; and orange shows dual mutants. Small group 1 with 9 dual mutants and large group 2 with 92 dual mutants are indicated. The NA sequence and corresponding HA of each virus are connected by lines. Group 1 dual mutants have HA 5a.2a_C.1. Only 2 group 2 dual mutants (A/British Columbia/PHL1108/2023 collected in May 2023 and A/France/IDF-RELAB-IPP24993/2023 collected in October 2023) have HA 5a.2a_C.1; remaining group 2 dual mutants shared HA 5a.2a.1_C.1.1.1. HA 5a.2a_C.1 is represented by A/Sydney/5/2021, the Southern Hemisphere 2023 vaccine egg/cell prototype virus. HA 5a.2a.1_C.1.1.1 is represented by A/Victoria/4897/2022, the Northern Hemisphere 2023–2024 vaccine egg prototype virus. The Northern Hemisphere 2023–2024 vaccine cell prototype virus, A/Wisconsin/67/2022, represents HA 5a.2a.1_C.1.1. HA, hemagglutinin; NA, neuraminidase.

On the basis of NA phylogenetic analysis, we determined that most single S247N mutants belonged to either subclade C.5 (16%) or the most abundantly sequenced subclade, C.5.3 (80%) (*8*). All subclade C.5.3 viruses share substitution S200N located at the antibody binding family site VI (Figure 1, panel A). Most single I223V mutants (92%) also belonged to subclade C.5.3 and formed a distinct branch with substitution S366N located at the antibody binding family site III. Within that branch, 2 separate introductions of S247N have occurred, giving rise to dual I223V + S247N mutants that could be divided into distinct groups 1 and 2 (Figure 2). Only 9 dual mutants collected in 6 countries belonged to group 1 and shared an additional substitution R257K. Group 2 encompassed 92 dual mutants from countries with multiple detections (i.e., France, Netherlands, Bangladesh, the United Kingdom, Oman, and Niger) (Figure 1, panel B).

To further characterize the dual mutants, we performed hemagglutinin (HA) phylogenetic analysis. Two major HA clades, 6B.1A.5a.2a (5a.2a) and 6B.1A.5a.2a.1 (5a.2a.1), were seen globally during this period (*8*,*9*). Viruses belonging to HA subclades 5a.2a_C.1 (55%–61%) and 5a.2a.1_C.1.1.1 (13%–39%) predominated. Of note, all group 1 dual mutants had HA from 5a.2a_C.1, represented by the previous vaccine prototype virus A/Sydney/5/2021 (Figure 2). Conversely, most group 2 dual mutants had HA from 5a.2a.1_C.1.1.1, represented by the current vaccine prototype virus A/Victoria/4897/2022, and most shared 2 HA changes: R45K in HA1 and L2I in HA2 (Figure 2).

## Conclusions

We report the emergence and intercontinental spread of pH1N1 viruses displaying reduced susceptibility to oseltamivir resulting from acquisition of NA-I223V + S247N mutations. Emergence of the dual mutants was also recently noticed by researchers in Hong Kong (*10*). The dual mutants that we tested retained susceptibility to other approved influenza antiviral drugs, including baloxavir. Analysis of available sequence data revealed that dual mutants have been in global circulation since May 2023; overall detection frequency was low (0.67%, 101/15,003). However, those data may not necessarily represent the actual proportion of what was in circulation because of differences in surveillance and sequencing strategies in each country.

Substitutions at residues 223 or 247 were previously reported and occurred spontaneously in circulating viruses (*5*,*11*,*12*). pH1N1 viruses with S247N circulated in several countries during 2009–2011 (*11*), and influenza B viruses with I223V (I221V in B numbering) were found in several US states during 2010–2011 (*12*). Isoleucine at 223 is a highly conserved framework residue in the NA active site. The S247N substitution may alter the hydrogen bonding network of the active site and the conformation of the residue E277 side chain, thereby weakening oseltamivir binding (*11*). Changes at 223 or 247 are monitored because they can enhance drug resistance by combining with mutations at other residues (*11*,*13*,*14*).

Rapid spread of dual mutants to countries on different continents suggests no substantial loss in their replicative fitness and transmissibility. I223V was shown to alter NA activity (*14*), and change at 247 may produce a similar effect, which warrants the question whether signature substitution(s) of NA subclade C.5.3 and of the branch to which the dual mutants belong (i.e., S200N, S366N) could serve as prerequisites for emergence of dual mutants. All group 1 dual mutants had an additional substitution R257K, which was previously associated with restoring NA activity of viruses with the H275Y substitution (*15*). Conversely, most group 2 viruses acquired HA from subclade 5a.2a.1_C.1.1.1 by reassortment, which may have helped to restore the functional HA and NA balance. Acquisition of the antigenically distinct HA could further enhance the spread of group 2 dual mutants. Our study highlights the need to closely monitor evolution of dual mutants because additional changes may further affect susceptibility to antiviral drugs or provide a competitive advantage over circulating wild-type viruses.

Appendix Additional information for study of multicountry spread of influenza A(H1N1)pdm09 viruses with reduced oseltamivir inhibition, May 2023–February 2024.
