# Supplementary material for: Multicountry Spread of Influenza A(H1N1)pdm09 Viruses with Reduced Oseltamivir Inhibition, May 2023–February 2024
Source: Emerg Infect Dis. 2024 Jul;30(7):1410–5. doi: 10.3201/eid3007.240480 (PMC11210663; doi:10.3201/eid3007.240480)
Supplement: Appendix — Additional information for study of multicountry spread of influenza A(H1N1)pdm09 viruses with reduced oseltamivir inhibition, May 2023–February 2024. [file 24-0480-Techapp-s1.pdf]

Article DOI: https://doi.org/10.3201/eid3007.240480

EID cannot ensure accessibility for supplementary materials supplied by authors. Readers who have difficulty accessing supplementary content should contact the authors for assistance.

Multicountry Spread of Influenza A(H1N1)pdm09 Viruses with Reduced Oseltamivir Inhibition, May 2023–February 2024

Appendix

Appendix Table 1. Influenza A(H1N1)pdm09 viruses with amino acid substitutions in neuraminidase that may affect inhibition by oseltamivir: NGS-based virologic surveillance at CDC, May 2023 – February 2024*

Virus name	Collection Date	Patient's age/ gender	Country	HA clade _subclade	NA subclade	Epi Isolate Id
H275Y (n=4)						
A/Montana/53/2023	2023-12-05	72/F	USA	5a.2a_C.1	C.5	EPI_ISL_18742880
A/Panama/M271629/2023	2023-07-13	40/M	Panama	5a.2a_C.1	C.5.3	EPI_ISL_18304046
A/Argentina/3270/2023†	2023-06-26	47/M	Argentina	5a.2a_C.1	C.5.3	EPI_ISL_18669559
A/Iowa/34/2023‡	2023-06-12	unk	USA	5a.2a_C.1	C.5.3	EPI_ISL_18110274
I223V+S247N (n=17)						
A/Connecticut/11/2023	2023-10-14	2/F	USA	5a.2a_C.1	C.5.3	EPI_ISL_18586500
A/Bangladesh/2469/2023	2023-08-16	4/F	Bangladesh	5a.2a.1_C.1.1.1	C.5.3	EPI_ISL_18742789
A/Bangladesh/1607/2023	2023-09-02	3/M	Bangladesh	5a.2a.1_C.1.1.1	C.5.3	EPI_ISL_18742804
A/Bangladesh/2528/2023	2023-09-05	3/F	Bangladesh	5a.2a.1_C.1.1.1	C.5.3	EPI_ISL_18742795
A/Bangladesh/2478/2023	2023-09-16	3/F	Bangladesh	5a.2a.1_C.1.1.1	C.5.3	EPI_ISL_19091568
A/Bangladesh/2336/2023	2023-09-17	5/F	Bangladesh	5a.2a.1_C.1.1.1	C.5.3	EPI_ISL_19091569
A/Bangladesh/0017/2023	2023-09-18	2/M	Bangladesh	5a.2a.1_C.1.1.1	C.5.3	EPI_ISL_18857362
A/Bangladesh/3188/2023	2023-09-19	0/F	Bangladesh	5a.2a.1_C.1.1.1	C.5.3	EPI_ISL_18742820
A/Bangladesh/4019/2023	2023-09-21	70/M	Bangladesh	5a.2a.1_C.1.1.1	C.5.3	EPI_ISL_18857373
A/Bangladesh/9005/2023	2023-10-05	80/F	Bangladesh	5a.2a.1_C.1.1.1	C.5.3	EPI_ISL_18857353
A/Bangladesh/0012/2023	2023-10-14	62/M	Bangladesh	5a.2a.1_C.1.1.1	C.5.3	EPI_ISL_18857341
A/Bangladesh/1231014022/2023	2023-10-19	50/F	Bangladesh	5a.2a.1_C.1.1.1	C.5.3	EPI_ISL_18857367
A/Hong Kong/2591/2023	2023-10-03	1/F	Hong Kong	5a.2a_C.1	C.5.3	EPI_ISL_18785914
A/Maldives/1962/2023	2023-11-05	64/F	Maldives	5a.2a.1_C.1.1.1	C.5.3	EPI_ISL_18786049
A/Niger/6452/2023	2023-09-13	1/F	Niger	5a.2a.1_C.1.1.1	C.5.3	EPI_ISL_18607693
A/Niger/6526/2023	2023-09-14	20/F	Niger	5a.2a.1_C.1.1.1	C.5.3	EPI_ISL_18607655
A/Niger/6710/2023	2023-09-27	35/F	Niger	5a.2a.1_C.1.1.1	C.5.3	EPI_ISL_18607687
I223V (n=18)						
A/Pennsylvania/65/2023	2023-10-16	2/F	USA	5a.2a_C.1	C.5.3	EPI_ISL_18527404
A/Pennsylvania/64/2023	2023-10-16	5/M	USA	5a.2a_C.1	C.5.3	EPI_ISL_18527398
A/Michigan/69/2023	2023-11-14	8/F	USA	5a.2a_C.1	C.5.3	EPI_ISL_18742817
A/New York/74/2023	2023-12-30	4/F	USA	5a.2a_C.1.7	C.4	EPI_ISL_18862625
A/Abu Dhabi/7431/2023	2023-06-25	24/M	Abu Dhabi	5a.2a_C.1	C.5.3	EPI_ISL_18337973
A/Abu Dhabi/11211/2023	2023-10-02	23/F	Abu Dhabi	5a.2a_C.1	C.5.3	EPI_ISL_18527417
A/Abu Dhabi/11219/2023	2023-10-02	30/F	Abu Dhabi	5a.2a_C.1	C.5.3	EPI_ISL_18527430
A/Rak/11784/2023	2023-10-01	8/M	Abu Dhabi	5a.2a_C.1	C.5.3	EPI_ISL_18527422
A/Rak/11794/2023	2023-10-03	0/M	Abu Dhabi	5a.2a_C.1	C.5.3	EPI_ISL_18527340
A/Bangladesh/1855/2023	2023-08-12	0/F	Bangladesh	5a.2a_C.1	C.5.3	EPI_ISL_18742769
A/Bangladesh/4022/2023	2023-08-28	2/F	Bangladesh	5a.2a_C.1	C.5.3	EPI_ISL_18742759
A/Bangladesh/3157/2023	2023-09-02	16/M	Bangladesh	5a.2a_C.1	C.5.3	EPI_ISL_18742786
A/Bangladesh/4006/2023	2023-09-11	9/M	Bangladesh	5a.2a_C.1	C.5.3	EPI_ISL_18857350
A/Bangladesh/4001/2023	2023-10-02	0/M	Bangladesh	5a.2a_C.1	C.5.3	EPI_ISL_18857356
A/Bahrain/0044/2023	2023-09-13	77/M	Bahrain	5a.2a_C.1	C.5.3	EPI_ISL_18586472
A/Bahrain/5931/2023	2023-07-13	43/M	Bahrain	5a.2a_C.1	C.5.3	EPI_ISL_18143140

Virus name	Collection Date	Patient's age/ gender	Country	HA clade _subclade	NA subclade	Epi Isolate Id
A/Ontario/RV00632/2023	2023-06-05	unk	Canada	5a.2a_C.1	C.5.3	EPI_ISL_18143131
A/Costa Rica/0511/2023	2023-10-06	65/F	Costa Rica	5a.2a_C.1	C.5.3	EPI_ISL_18527495
			S247N (n=15)			
A/California/110/2023	2023-11-14	20/M	USA	5a.2a_C.1	C.5.3	EPI_ISL_18709269
A/Ohio/26/2023	2023-11-15	2/F	USA	5a.2a_C.1	C.5.3	EPI_ISL_18669531
A/Puerto Rico/14/2023	2023-11-29	50/F	USA	5a.2a.1_C.1.1.1	C.5.2	EPI_ISL_18785868
A/Missouri/35/2023	2023-12-11	72/M	USA	5a.2a.1_C.1.1.1	C.5.3	EPI_ISL_18857378
A/Colorado/93/2023	2023-12-14	73/F	USA	5a.2a.1_C.1.1.1	C.5.1.1	EPI_ISL_18785863
A/District of Columbia/38/2023	2023-12-27	unk	USA	5a.2a.1_C.1.1	C.5.3	EPI_ISL_18862824
A/Abu Dhabi/11977/2023	2023-10-12	40/M	Abu Dhabi	5a.2a_C.1	C	EPI_ISL_18527442
A/Abu Dhabi/12014/2023	2023-10-13	7/M	Abu Dhabi	5a.2a_C.1	C.5	EPI_ISL_18527426
A/Amapa/2023-015904-IEC/2023	2023-05-10	2/M	Brazil	5a.2a_C.1	C.5.3	EPI_ISL_18091714
A/Bhutan/2546/2023	2023-09-06	54/F	Bhutan	5a.2a_C.1	C.5	EPI_ISL_18607702
A/Bhutan/2581/2023	2023-09-20	6/F	Bhutan	5a.2a_C.1	C.5	EPI_ISL_18607689
A/Bhutan/2582/2023	2023-09-21	4/M	Bhutan	5a.2a_C.1	C.5	EPI_ISL_18607667
A/Manitoba/RV01247/2023	2023-11-06	unk	Canada	5a.2a_C.1	C.5	EPI_ISL_18785999
A/Hong Kong/1509/2023	2023-07-09	1/M	Hong Kong	5a.2a_C.1	C.5.3	EPI_ISL_18143051
A/Oman/0084/2023	2023-07-12	22/F	Oman	5a.2a_C.1	C.5	EPI_ISL_18304020

*Summary of NA amino acid substitutions assessed for their effects on inhibition by NA inhibitors prepared by members of WHO-Antiviral Working Group (https://cdn.who.int/media/docs/default-source/global-influenza-programme/1.-nai_human_reduced-susceptibility-marker-table-(who)_07.03.23_update.pdf) was used as a reference for NA sequence analysis. A total of 2,039 NA sequences of A(H1N1)pdm09 viruses (duplicate sequences excluded; n=1,274 viruses collected from the US and n=765 from 38 other countries) were analyzed to screen for previously listed or suspected NA amino acid substitutions. Substitutions are shown according to a subtype-specific NA amino acid numbering system.
†This virus also contains NA-S247N. Virus was not recovered in cell culture.
‡This virus contains H275Y/H + D199N/D mixture.

Appendix Table 2. Oseltamivir IC_{50} and fold-change of available A(H1N1)pdm09 virus isolates with dual NA-I223V+S247N substitutions compared to sequence-matched wildtype virus*

Virus name	HA clade_subclade	NA change vs A/Wisconsin/67/2022†: Residues 223 and 247	NA change vs A/Wisconsin/67/2022†: NA subclade C.5.3 specific	NA change vs A/Wisconsin/67/2022†: Additional	Oseltamivir IC_{50} nM, mean ± SD (fold)‡
Test viruses					
A/Bangladesh/0017/2023	5a.2a.1_C.1.1.1	I223V + S247N	V13I, S200N, L339S, S366N	-	3.05 ± 0.26 (16)
A/Bangladesh/1231014022/2023	5a.2a.1_C.1.1.1			-	2.71 ± 0.33 (14)
A/Bangladesh/9005/2023	5a.2a.1_C.1.1.1			T188I	2.58 ± 0.52 (14)
A/Niger/6452/2023	5a.2a.1_C.1.1.1			S286G	2.70 ± 0.28 (14)
A/Hong Kong/2591/2023	5a.2a_C.1			R257K	2.41 ± 0.34 (13)
A/Connecticut/11/2023	5a.2a_C.1			S79P, R257K, M269V	2.80 ± 0.42 (15)
Reference virus					
A/Togo/0706/2023	5a.2a_C.1	I223 + S247 (WT)	V13I, S200N, L339S, S366N	-	0.19 ± 0.04

NA: neuraminidase; SD: standard deviation
*NGS-flagged A(H1N1)pdm09 viruses were propagated in MDCK-SIAT1 cells, followed by sequence confirmation of virus isolates. Oseltamivir susceptibility of available virus isolates with dual NA-I223V+S247N substitutions were tested using CDC-standardized fluorescence-based NA inhibition assay. Virus isolates were tested in at least 2-3 independent tests and average ± SD of IC_{50}s is shown. Fold changes in IC_{50}s of test viruses were calculated compared to IC_{50} of a NA sequence-matched wildtype virus (A/Togo/0706/2023). NA sequence of A/Togo/0706/2023 is exact match to two tested dual mutant viruses (A/Bangladesh/0017/2023 and A/Bangladesh/1231014022/2023). The other four dual mutants (A/Bangladesh/9005/2023, A/Niger/6452/2023, A/Hong Kong/2591/2023, and A/Connecticut/11/2023) differ at 1-3 additional amino acids compared to A/Togo/0706/2023 virus.
†NA sequence changes of test and reference viruses were shown in comparison to A/Wisconsin/67/2022, the northern hemisphere 2023-2024 vaccine cell prototype virus for A(H1N1)pdm09 component. NA of all the test and reference viruses belong to NA subclade C.5.3.
‡Fold changes were interpreted according to WHO classification criteria: for type A: <10-fold – normal inhibition; 10-100-fold – reduced inhibition; >100-fold – highly reduced inhibition.

Appendix Table 3. List of A(H1N1)pdm09 viruses carrying dual NA substitutions I223V+S247N, May 2023 - February 2024*

Patient location		Virus name	Collection Date	NA group†	HA clade _subclade	Patient's age/gender	GISAID EpiFlu^{tm} Epi Isolate Id (EPI_ISL_)	Originating laboratory	Submitting laboratory
Africa	Ethiopia	A/Ethiopia/AA08281/2023	2023/09/25	2	5a.2a.1_C.1.1.1	unk	18714797	Ethiopian Public Health Institute, National Influenza Laboratory	Ethiopian Public Health Institute
	Niger	A/Niger/6452/2023	2023/09/13	2	5a.2a.1_C.1.1.1	1/F	18607693	Centre de Recherche Medicale et Sanitaire (CERMES)	Centers for Disease Control and Prevention
		A/Niger/6526/2023	2023/09/14	2	5a.2a.1_C.1.1.1	20/F	18607655	Centre de Recherche Medicale et Sanitaire (CERMES)	Centers for Disease Control and Prevention
		A/Niger/6710/2023	2023/09/27	2	5a.2a.1_C.1.1.1	35/F	18607687	Centre de Recherche Medicale et Sanitaire (CERMES)	Centers for Disease Control and Prevention
Asia	Bangladesh	A/Bangladesh/2469/2023	2023/08/16	2	5a.2a.1_C.1.1.1	4/F	18742789	Institute of Epidemiology, Disease Control and Research	Centers for Disease Control and Prevention
		A/Bangladesh/1607/2023	2023/09/02	2	5a.2a.1_C.1.1.1	3/M	18742804	Institute of Epidemiology, Disease Control and Research	Centers for Disease Control and Prevention
		A/Bangladesh/2528/2023	2023/09/05	2	5a.2a.1_C.1.1.1	3/F	18742795	Institute of Epidemiology, Disease Control and Research	Centers for Disease Control and Prevention
		A/Bangladesh/2478/2023	2023/09/16	2	5a.2a.1_C.1.1.1	3/F	19091568	Institute of Epidemiology, Disease Control and Research	Centers for Disease Control and Prevention
		A/Bangladesh/2336/2023	2023/09/17	2	5a.2a.1_C.1.1.1	5/M	19091569	Institute of Epidemiology, Disease Control and Research	Centers for Disease Control and Prevention
		A/Bangladesh/0017/2023	2023/09/18	2	5a.2a.1_C.1.1.1	2/M	18857362	International Centre for Diarrhoeal Disease Research, Bangladesh	Centers for Disease Control and Prevention
		A/Bangladesh/3188/2023	2023/09/19	2	5a.2a.1_C.1.1.1	0/F	18742820	Institute of Epidemiology, Disease Control and Research	Centers for Disease Control and Prevention
		A/Bangladesh/4019/2023	2023/09/21	2	5a.2a.1_C.1.1.1	70/M	18857373	International Centre for Diarrhoeal Disease Research, Bangladesh	Centers for Disease Control and Prevention

Patient location	Virus name	Collection Date	NA group†	HA clade _subclade	Patient's age/gender	GISAID EpiFlu^{tm} Epi Isolate Id (EPI_ISL_)	Originating laboratory	Submitting laboratory
	A/Bangladesh/9005/2023	2023/10/05	2	5a.2a.1_C.1.1.1	80/F	18857353	International Centre for Diarrhoeal Disease Research, Bangladesh	Centers for Disease Control and Prevention
	A/Bangladesh/0012/2023	2023/10/14	2	5a.2a.1_C.1.1.1	62/M	18857341	International Centre for Diarrhoeal Disease Research, Bangladesh	Centers for Disease Control and Prevention
	A/Bangladesh/1231014022/2023	2023/10/19	2	5a.2a.1_C.1.1.1	50/F	18857367	International Centre for Diarrhoeal Disease Research, Bangladesh	Centers for Disease Control and Prevention
Hong Kong	A/Hong Kong/2591/2023	2023/10/03	1	5a.2a_C.1	1/F	18785914	Centre for Health Protection	Centers for Disease Control and Prevention
	A/Hong Kong/HKU-231217-085/2023	2023/10/08	1	5a.2a_C.1	unk	18671990	The University of Hong Kong	The University of Hong Kong, Center of Influenza Research
	A/Hong Kong/HKU-231217-087/2023	2023/10/21	1	5a.2a_C.1	unk	18671992	The University of Hong Kong	The University of Hong Kong, Center of Influenza Research
	A/Hong Kong/HKU-231217-088/2023	2023/10/21	1	5a.2a_C.1	unk	18671993	The University of Hong Kong	The University of Hong Kong, Center of Influenza Research
Maldives	A/Maldives/1962/2023	2023/11/05	2	5a.2a.1_C.1.1.1	64/F	18786049	Ministry of Health Maldives	Centers for Disease Control and Prevention
Oman	A/Salalah/52326095/2023	2023/09/10	2	5a.2a.1_C.1.1.1	16/M	18557972	Central Public Health Laboratory, Ministry of Health (Crick)	Crick Worldwide Influenza Centre
	A/Oman/CPHL_52330055/2023	2023/10/07	2	5a.2a.1_C.1.1.1	6Mon/M	18544342	Central Public Health Laboratories Oman	Central Public Health Laboratories Oman
	A/Oman/CPHL_7234967/2023	2023/10/08	2	5a.2a.1_C.1.1.1	19/F	18544343	Central Public Health Laboratories Oman	Central Public Health Laboratories Oman

Patient location		Virus name	Collection Date	NA group†	HA clade _subclade	Patient's age/gender	GISAID EpiFlu[tm] Epi Isolate Id (EPI_ISL_)	Originating laboratory	Submitting laboratory
		A/Oman/CPHL_7235240/2023	2023/10/16	2	5a.2a.1_C.1.1.1	65/M	18544353	Central Public Health Laboratories Oman	Central Public Health Laboratories Oman
		A/Oman/CPHL_7235304/2023	2023/10/18	2	5a.2a.1_C.1.1.1	6Mon/M	18544358	Central Public Health Laboratories Oman	Central Public Health Laboratories Oman
		A/Oman/CPHL_7236077/2023	2023/11/02	2	5a.2a.1_C.1.1.1	43/F	18716431	Central Public Health Laboratories Oman	Central Public Health Laboratories Oman
		A/Oman/CPHL_7236187/2023	2023/11/06	2	5a.2a.1_C.1.1.1	21/M	18716444	Central Public Health Laboratories Oman	Central Public Health Laboratories Oman
		A/Oman/CPHL_7236254/2023	2023/11/08	2	5a.2a.1_C.1.1.1	21/F	18716446	Central Public Health Laboratories Oman	Central Public Health Laboratories Oman
		A/Oman/CPHL_7236721/2023	2023/11/19	2	5a.2a.1_C.1.1.1	16/F	18716442	Central Public Health Laboratories Oman	Central Public Health Laboratories Oman
Europe	France	A/Paris/16713/2023	2023/07/19	2	5a.2a.1_C.1.1.1	46/F	18131735	Institut Pasteur	Institut Pasteur
		A/France/IDF-RELAB-IPP24993/2023	2023/10/13	2	5a.2a_C.1	81/M	18718249	Institut Pasteur	Institut Pasteur
		A/France/ARA-HCL023165654501/2023	2023/10/20	2	5a.2a.1_C.1.1.1	70/F	18585235	CNR Virus des Infections Respiratoires - France SUD	CNR Virus des Infections Respiratoires, France SUD
		A/France/BRE-RELAB-IPP29645/2023	2023/11/21	2	5a.2a.1_C.1.1.1	49/M	18731062	Institut Pasteur	Institut Pasteur
		A/France/BRE-RELAB-IPP30709/2023	2023/12/02	2	5a.2a.1_C.1.1.1	34/F	18731165	Institut Pasteur	Institut Pasteur
		A/France/HDF-IPP31144/2023	2023/12/06	2	5a.2a.1_C.1.1.1	83/F	18731168	Institut Pasteur	Institut Pasteur
		A/France/ARA-HCL023192539401/2023	2023/12/07	2	5a.2a.1_C.1.1.1	38/M	18795127	CNR Virus des Infections Respiratoires - France SUD	CNR Virus des Infections Respiratoires, France SUD
		A/France/BRE-RELAB-IPP33064/2023	2023/12/11	2	5a.2a.1_C.1.1.1	52/M	18731254	Institut Pasteur	Institut Pasteur
		A/France/IDF-RELAB-IPP33027/2023	2023/12/12	2	5a.2a.1_C.1.1.1	unk	18731286	Institut Pasteur	Institut Pasteur
		A/France/NAQ-RELAB-HCL023200875501/2023	2023/12/12	2	5a.2a.1_C.1.1.1	56/F	18795240	CNR Virus des Infections Respiratoires - France SUD	CNR Virus des Infections Respiratoires, France SUD

Patient location	Virus name	Collection Date	NA group†	HA clade _subclade	Patient's age/gender	GISAID EpiFlu^{tm} Epi Isolate Id (EPI_ISL_)	Originating laboratory	Submitting laboratory
	A/France/IDF-RELAB-IPP32967/2023	2023/12/13	2	5a.2a.1_C.1.1.1	3/F	18731230	Institut Pasteur	Institut Pasteur
	A/France/NAQ-RELAB-HCL023205011801/2023	2023/12/21	2	5a.2a.1_C.1.1.1	72/F	18795403	CNR Virus des Infections Respiratoires - France SUD	CNR Virus des Infections Respiratoires, France SUD
	A/France/NAQ-RELAB-HCL023205017701/2023	2023/12/22	2	5a.2a.1_C.1.1.1	16/F	18795425	CNR Virus des Infections Respiratoires - France SUD	CNR Virus des Infections Respiratoires, France SUD
	A/France/ARA-HCL023203765401/2023	2023/12/27	2	5a.2a.1_C.1.1.1	35/F	18795463	CNR Virus des Infections Respiratoires - France SUD	CNR Virus des Infections Respiratoires, France SUD
	A/France/IDF-RELAB-IPP00942/2024	2023/12/27	2	5a.2a.1_C.1.1.1	59/M	18852686	Institut Pasteur	Institut Pasteur
	A/France/ARA-HCL023205241401/2023	2023/12/29	2	5a.2a.1_C.1.1.1	29/F	18795537	CNR Virus des Infections Respiratoires - France SUD	CNR Virus des Infections Respiratoires, France SUD
	A/France/ARA-HCL023205715501/2023	2023/12/31	2	5a.2a.1_C.1.1.1	unk/F	18795476	CNR Virus des Infections Respiratoires - France SUD	CNR Virus des Infections Respiratoires, France SUD
	A/France/NAQ-RELAB-HCL024006387101/2024	2024/01/03	2	5a.2a.1_C.1.1.1	21/F	18855796	CNR Virus des Infections Respiratoires - France SUD	CNR Virus des Infections Respiratoires, France SUD
	A/France/NAQ-RELAB-HCL024006386101/2024	2024/01/05	2	5a.2a.1_C.1.1.1	69/M	18855793	CNR Virus des Infections Respiratoires - France SUD	CNR Virus des Infections Respiratoires, France SUD
	A/France/NAQ-RELAB-HCL024009907601/2024	2024/01/09	2	5a.2a.1_C.1.1.1	73/M	18926375	CNR Virus des Infections Respiratoires - France SUD	CNR Virus des Infections Respiratoires, France SUD
	A/France/NAQ-RELAB-HCL024009905601/2024	2024/01/10	2	5a.2a.1_C.1.1.1	78/M	18885619	CNR Virus des Infections Respiratoires - France SUD	CNR Virus des Infections Respiratoires, France SUD
	A/France/NAQ-RELAB-HCL024014240801/2024	2024/01/15	2	5a.2a.1_C.1.1.1	41/M	18885695	CNR Virus des Infections Respiratoires - France SUD	CNR Virus des Infections Respiratoires, France SUD
	A/France/NAQ-RELAB-HCL024014250501/2024	2024/01/17	2	5a.2a.1_C.1.1.1	88/M	18885753	CNR Virus des Infections Respiratoires - France SUD	CNR Virus des Infections Respiratoires, France SUD
	A/France/ARA-HCL024017186601/2024	2024/01/30	2	5a.2a.1_C.1.1.1	23/F	18926492	CNR Virus des Infections Respiratoires - France SUD	CNR Virus des Infections

Patient location	Virus name	Collection Date	NA group†	HA clade _subclade	Patient's age/gender	GISAID EpiFlu^{tm} Epi Isolate Id (EPI_ISL_)	Originating laboratory	Submitting laboratory
								Respiratoires, France SUD
Netherlands	A/Netherlands/01698/2023	2023/09/26	1	5a.2a_C.1	unk/F	18373309	Erasmus Medical Center	Erasmus Medical Centre
	A/Netherlands/10558/2023	2023/11/05	2	5a.2a.1_C.1.1.1	81/M	18545930	Academic Medical Center, University of Amsterdam	National Institute for Public Health and the Environment
	A/Netherlands/10575/2023	2023/12/07	2	5a.2a.1_C.1.1.1	43/M	18652672	National Institute for Public Health and the Environment (RIVM)	National Institute for Public Health and the Environment
	A/Netherlands/02111/2023	2023/12/16	2	5a.2a.1_C.1.1.1	unk/F	18746132	Erasmus Medical Center	Erasmus Medical Centre
	A/Netherlands/10631/2023	2023/12/20	2	5a.2a.1_C.1.1.1	57/F	18718235	National Institute for Public Health and the Environment (RIVM)	National Institute for Public Health and the Environment
	A/Netherlands/03127/2023	2023/12/30	2	5a.2a.1_C.1.1.1	unk/F	18790233	Erasmus Medical Center	Erasmus Medical Centre
	A/Netherlands/00101/2024	2024/01/04	2	5a.2a.1_C.1.1.1	unk/F	18790241	Erasmus Medical Center	Erasmus Medical Centre
	A/Netherlands/00088/2024	2024/01/05	2	5a.2a.1_C.1.1.1	unk/F	18790231	Erasmus Medical Center	Erasmus Medical Centre
	A/Netherlands/10037/2024	2024/01/10	2	5a.2a.1_C.1.1.1	43/F	18773040	National Institute for Public Health and the Environment (RIVM)	National Institute for Public Health and the Environment
	A/Netherlands/10144/2024	2024/01/15	2	5a.2a.1_C.1.1.1	unk	18877868	National Institute for Public Health and the Environment (RIVM)	National Institute for Public Health and the Environment
	A/Netherlands/10125/2024	2024/01/17	2	5a.2a.1_C.1.1.1	40/F	18842152	National Institute for Public Health and the Environment (RIVM)	National Institute for Public Health and the Environment
	A/Netherlands/00330/2024	2024/01/20	2	5a.2a.1_C.1.1.1	unk	18873548	Erasmus Medical Center	Erasmus Medical Centre

Patient location	Virus name	Collection Date	NA group†	HA clade _subclade	Patient's age/gender	GISAID EpiFlu^{tm} Epi Isolate Id (EPI_ISL_)	Originating laboratory	Submitting laboratory
	A/Netherlands/00257/2024	2024/01/22	2	5a.2a.1_C.1.1.1	unk/F	18854117	Erasmus Medical Center	Erasmus Medical Centre
	A/Netherlands/00393/2024	2024/01/22	2	5a.2a.1_C.1.1.1	unk	18873568	Erasmus Medical Center	Erasmus Medical Centre
	A/Netherlands/00315/2024	2024/01/23	2	5a.2a.1_C.1.1.1	unk	18873544	Erasmus Medical Center	Erasmus Medical Centre
	A/Netherlands/10172/2024	2024/01/23	2	5a.2a.1_C.1.1.1	46/F	18877908	National Institute for Public Health and the Environment (RIVM)	National Institute for Public Health and the Environment
	A/Netherlands/10231/2024	2024/01/25	2	5a.2a.1_C.1.1.1	unk	18877886	National Institute for Public Health and the Environment (RIVM)	National Institute for Public Health and the Environment
	A/Netherlands/10264/2024	2024/01/25	2	5a.2a.1_C.1.1.1	unk	18890414	National Institute for Public Health and the Environment (RIVM)	National Institute for Public Health and the Environment
	A/Netherlands/10240/2024	2024/01/25	2	5a.2a.1_C.1.1.1	57/M	18877926	National Institute for Public Health and the Environment (RIVM)	National Institute for Public Health and the Environment
	A/Netherlands/00507/2024	2024/01/26	2	5a.2a.1_C.1.1.1	unk/M	18854137	Erasmus Medical Center	Erasmus Medical Centre
	A/Netherlands/00474/2024	2024/01/28	2	5a.2a.1_C.1.1.1	unk	18873591	Erasmus Medical Center	Erasmus Medical Centre
	A/Netherlands/10286/2024	2024/01/29	2	5a.2a.1_C.1.1.1	53/M	18890469	National Institute for Public Health and the Environment (RIVM)	National Institute for Public Health and the Environment
	A/Netherlands/10430/2024	2024/02/08	2	Not available	67/F	18913313	National Institute for Public Health and the Environment (RIVM)	National Institute for Public Health and the Environment
	A/Netherlands/00707/2024	2024/01/30	2	5a.2a.1_C.1.1.1	unk	18918723	Erasmus Medical Center	Erasmus Medical Centre

Patient location	Virus name	Collection Date	NA group†	HA clade _subclade	Patient's age/gender	GISAID EpiFlu[tm] Epi Isolate Id (EPI_ISL_)	Originating laboratory	Submitting laboratory
	A/Netherlands/00901/2024	2024/02/15	2	5a.2a.1_C.1.1.1	unk	18942004	Erasmus Medical Center	Erasmus Medical Centre
	A/Netherlands/10463/2024	2024/02/12	2	5a.2a.1_C.1.1.1	unk	18936083	National Institute for Public Health and the Environment (RIVM)	National Institute for Public Health and the Environment
	A/Netherlands/10481/2024	2024/02/12	2	5a.2a.1_C.1.1.1	51/M	18936126	National Institute for Public Health and the Environment (RIVM)	National Institute for Public Health and the Environment
	A/Netherlands/00988/2024	2024/02/21	2	5a.2a.1_C.1.1.1	unk	18961731	Erasmus Medical Center	Erasmus Medical Centre
	A/Netherlands/01137/2024	2024/02/22	2	5a.2a.1_C.1.1.1	unk	18961761	Erasmus Medical Center	Erasmus Medical Centre
	A/Netherlands/10506/2024	2024/02/20	2	5a.2a.1_C.1.1.1	35/F	18950653	National Institute for Public Health and the Environment (RIVM)	National Institute for Public Health and the Environment
Norway	A/Norway/10938/2023	2023/10/28	1	5a.2a_C.1	87/M	18567817	St. Olavs Hospital HF, Dept. of Medical Microbiology; Norwegian Institute of Public Health	Norwegian Institute of Public Health
Spain	A/Badajoz/18615008/2024	2024/01/04	2	5a.2a.1_C.1.1.1	unk	18854222	Hospital Universitario de Badajoz	Hospital Universitario de Badajoz
	A/Badajoz/18615018/2024	2024/01/04	2	5a.2a.1_C.1.1.1	unk	18854223	Hospital Universitario de Badajoz	Hospital Universitario de Badajoz
Sweden	A/Vasteras/2/2023	2023/09/26	1	5a.2a_C.1	52/F	18462073	Region Vastmanland, Laboratoriemedicin, Klinisk Mikrobiologi	Public Health Agency Sweden
United Kingdom	A/England/234680154/2023	2023/11/15	2	5a.2a.1_C.1.1.1	unk	18893536	UK Health Security Agency - Colindale	UKHSA / Respiratory Virus Unit
	A/England/234720236/2023	2023/11/17	2	5a.2a.1_C.1.1.1	unk	18893540	UK Health Security Agency - Colindale	UKHSA / Respiratory Virus Unit
	A/England/234740855/2023	2023/11/22	2	5a.2a.1_C.1.1.1	unk	18893544	UK Health Security Agency - Colindale	UKHSA / Respiratory Virus Unit
	A/United Kingdom/UO-6_e2fab162/2023	2023/12/12	2	Not available	unk	18873835	Unk/Imported into GISAID	Imported into GISAID
	A/United Kingdom/14475/2023	2023/12/19	2	5a.2a.1_C.1.1.1	13/M	18864718	U.S. Air Force School of Aerospace Medicine	U.S. Air Force School

Patient location		Virus name	Collection Date	NA group†	HA clade _subclade	Patient's age/gender	GISAID EpiFlu^{tm} Epi Isolate Id (EPI_ISL_)	Originating laboratory	Submitting laboratory
									of Aerospace Medicine
		A/England/240160509/2023	2023/12/23	2	5a.2a.1_C.1.1.1	unk	18873506	UK Health Security Agency - Colindale	UK Health Security Agency - Colindale
		A/England/240220547/2024	2024/01/09	1	5a.2a_C.1	unk	18873602	UK Health Security Agency - Colindale	UK Health Security Agency - Colindale
		A/Cardiff/1245/2024	2024/02/01	2	5a.2a.1_C.1.1.1	32/M	18944454	Public Health Wales Microbiology Cardiff	Public Health Wales Microbiology Cardiff
		A/England/240600978/2024	2024/01/05	2	5a.2a.1_C.1.1.1	unk	18962028	UK Health Security Agency - Colindale	UKHSA / Respiratory Virus Unit
North America	Canada	A/British_Columbia/PHL-1108/2023	2023/05/29	2	5a.2a_C.1	unk	18665767	B.C. Centre for Disease Control	Public Health Agency of Canada, National Microbiology Laboratory
	United States	A/Connecticut/11/2023	2023/10/14	1	5a.2a_C.1	2/F	18586581	Dr. Katherine A. Kelly Public Health Laboratory, Connecticut Department of Public Health	Centers for Disease Control and Prevention
		A/Michigan/UM-RR058315651/2023	2023/12/30	2	5a.2a.1_C.1.1.1	unk	18911182	University of Michigan, Lauring Lab, Department of Microbiology and Immunology	Imported into GISAID
Oceania	Australia	A/Perth/562/2023	2023/11/08	2	5a.2a.1_C.1.1.1	72/F	18955353	Pathwest QE II Medical Centre	Victorian Infectious Diseases Reference Laboratory
		A/Perth/614/2023	2023/11/08	2	5a.2a.1_C.1.1.1	72/F	18876659	Pathwest QE II Medical Centre	Victorian Infectious Diseases Reference Laboratory

*A total of 15,003 NA sequences of A(H1N1)pdm09 viruses (duplicate sequences excluded; ~15% from CDC surveillance and remaining ~85% from GISAID EpiFlu^{tm}) were analyzed to screen for amino acid substitutions at residues 223 and 247. Sequences from GISAID EpiFlu^{tm} accessed on March 11, 2024.
†NA of all dual mutant viruses belonged to subclade C.5.3. Dual mutants were divided into two groups based on their NA sequence difference. Group 1 shared additional substitution R257K than group 2.
